# Changes in Renal Parameters during a Training Camp among Handball Players in the Sub-Saharan Environment

**DOI:** 10.1155/2020/6031763

**Published:** 2020-02-20

**Authors:** Brigitte A. Tonon, Issiako Bio Nigan, Bruno Agboton, Polycarpe Gouthon, Basile Nouatin, Hippolyte Agboton

**Affiliations:** ^1^Laboratory Sports Performance, Health and Evaluation, National Institute of Youth, Physical Education and Sport (INJEPS), University of Abomey-Calavi (UAC), 01 P. O. Box 169, Porto-Novo, Benin; ^2^Department of Nephrology, National Teaching Hospital of Cotonou, Faculty of Health Science, University of Abomey-Calavi (UAC), 01 P. O. Box 827, Cotonou, Benin; ^3^Department of Cardiology, National Teaching Hospital of Cotonou, University of Abomey-Calavi (UAC), 01 P. O. Box 827, Cotonou, Benin

## Abstract

The aim of the study was to describe the changes in kidney parameters induced by 10 days of tapering (TP) during a training camp (TC), where the players were preparing for a group competition, in 15 female handball team members of a Division 1 Amateur of Benin, in the sub-Saharan environment. Measures were taken in all the players before and after the intensive training (IT) and tapering (TP) phases in an intervention study. The estimated glomerular filtration rate (eGFR) with the CKD-EPI 4-level race formula, the fractional excretions of sodium (FeNa) and potassium (FeK), the urine potassium-to-sodium ratio (Na/K urine), and the hemoglobin rate [Hb] were determined for all participants. At the end of IT, eGFR and FeNa increased, respectively, by 22.39% (*P* < 0.01) and 143.85% (*P* < 0.01), but the variation of FeK is not significant (*P* > 0.05). The number of abnormally low eGFR values (<90 mL/min/1.73 m^2^) was reduced from 11 to 5 (*P* < 0.05). At the end of TP, the eGFR and urine Na-to-K ratio remained on average constant (*P* > 0.05) but FeNa decreased by 96.32% (*P* < 0.001) and FeK increased by 144.41% (*P* < 0.001). The [Hb] rate increased by 9.80% (*P* < 0.001), and players had inadequate hydration practice. The results suggested that in addition to its already known effects, TP preserves the positive effects of IT on glomerular function in athletes preparing for a competition that presents a major challenge.

## 1. Introduction

The preparation of athletes for a sports competition is increasingly based on the organization of training camps, the last part of which consists of a tapering block that succeeds that of intensive training. Intensive training aims to optimize physical and physiological adaptations, and tapering is used to reduce accumulated fatigue, while maintaining the adaptations acquired [1, 2]. Training intensity should be high throughout the training camp, but the volume and/or training frequency should be reduced by 30 to 90% during tapering [3, 4]. It has been reported that this strategy has positive effects on the reconstitution of energy stocks, and muscular and psychological recovery [5, 6]. Recent studies have also highlighted the benefits of this strategy on physical performance in team sports [7, 8].

It has been recognized that renal function is strongly influenced by high exercise intensities, which cause profound changes in renal hemodynamic function, and electrolyte and protein excretion in people exposed to such conditions [9]. It is therefore reasonable to think that intensive training can induce, over a given period, significant disturbances in kidney function. Studies have been conducted on immediate renal adjustments during physical exercise [9, 10], but little work has been conducted on the effects of hot environment training on kidney function. Data on the long-term adaptation of athletes' kidneys to training have focused on renal blood flow and glomerular function, but most of these data have been collected in temperate climates [11, 12]. These data should be supplemented by others, especially those related to adaptations of the tubular function of athletes practicing in the warm atmosphere of sub-Saharan Africa.

In players of team sports such as handball, exposure to relatively high thermal stresses of the sub-Saharan environment, higher training loads, and the physiological demands of games, the kidney risk incurred could be significant. Only adequate physical preparation that combines intensive training and tapering can allow players to meet these requirements [1]. The magnitude of the risk incurred during these two phases of physical preparation in team sports in a hot environment is not known and must be determined. The present study aimed to describe in handball players, changes in glomerular and tubular parameters, induced by 10 days of tapering, preparatory to the final phase of the Benin Division 1 championship.

## 2. Materials and Methods

### 2.1. Protocol of the Study

**Figure 1:**
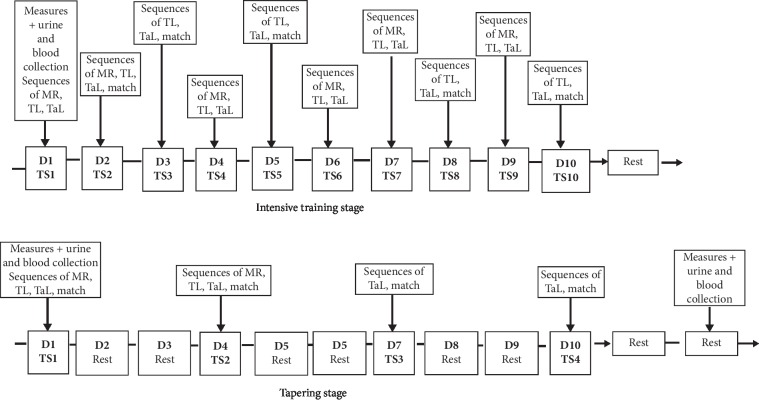
Design and context of the training camp. MR: muscular reinforcement; TL: technical learning; TaL: tactical learning; D: day; TS: training session.

This was an interventional study conducted among senior category players qualified for the final phase of the Division 1 handball championship of Benin. They participated in a training camp (TC) of ultimate preparation, consisting of an intensive training phase (IT) with high physical loads followed by tapering (TP) on the handball ground of the Stadium of Porto-Novo (Benin). On the first day of the TC (M1), 36 hours after its end (M2) and that of TP (M3), various measurements were taken in all the players (Figure 1). The study was carried out, following the ethical principles of Helsinki (1964) modified in 2013 [13], concerning the ethical principles. The informed and written consent of each player was obtained prior to participating in the study. The approval of the Scientific Committee for Science and Technology of Physical and Sports Activities (CSS/STAPS) of the University of Abomey-Calavi (UAC) was required and obtained (CSS/STAPS, N°001/CSS-STAPS/Pdt/07/12/11).

### 2.2. Participants

The study sample consisted of 15 black female handball players aged 19 to 38 years, including eight international players, who have competed in more than three African Champion Clubs' Cups. Only women in the senior category who played during the championship preliminaries for the targeted team participated in the study. Any player under antimalarial drug treatment, or any other medications that may influence or affect renal parameters, was not included in the study. A player who was absent for more than three of the ten IT training sessions, or more than one of the four TP training sessions, was excluded from the study.

### 2.3. Intervention

The 20-day training camp (Figure 1) consisted of a 10-day intensive training (IT) phase, followed by 10-day tapering (TP). The IT phase consisted of 10 high-intensity training sessions, one daily session, organized as (1) six sessions of 90 min, including 5 with 10 min of global play across the field; (2) two 90-min sessions, including 30-min of global play across the field; and (3) two handball games played in two 30-min sessions with a 10-min break. During the tapering phase, four training sessions were organized in 10 days, with one session every 72 hours (Figure 1). The four sessions of the TP, with high-intensity sequences, were organized in the same way as those of the IT phase, but the contents varied. Overall, the training sessions consisted of the following sequences:

- 10-min warm up at an intensity between 50 and 70% of the theoretical HRmax.
- 1 training sequence of maximum intensity speed races over 25 m in 5 to 15 min, for 1 to 2 series of 6 passages each, with 5-min rest between series.
- 1 technical training sequence of 12 to 30 min, composed of 2 to 3 series of 40 fast passes at maximum speed, in groups of 2 or 3 players, then 5 to 7 min of shots at the post and goalkeepers' parries in 1 to 2 series, at 90% of the theoretical HRmax, from 5 to 10 min of combinations of dribbles + 3 steps of momentum + shooting in suspension, at an intensity of 90 to 95% of the theoretical HRmax; and 5 min of defensive displacements in fundamental position, in 3 series with 30 s of rest between the series. The intensities varied from 90 to 95% of the theoretical HRmax.
- 1 tactical training sequence of 20 to 40 min, consisting of ball circulation with 5 or 6 players in attack placed, in 2 series of 10 repetitions with 30 s of rest between series, at an intensity of 90 to 95% theoretical HRmax. There were then rapid ball climbs with 3, 4, or 5 players, in 2 series for 3 or 5 min, with 1-min rest between series, at intensities of 90 to 95% of the theoretical HRmax.
- 2 global game sequences of 5 to 30 min over the whole field, with an application of the day's learning, at an intensity varying from 85 to 90% of the theoretical HRmax or.
- 30 min × 2 of theme match, with 10-min rest, at intensities of 80 to 85% of the theoretical HRmax.

### 2.4. Samples and Measurements

Samples of 10 mL of venous blood were collected at the cubital fold of the left elbow, in EDTA tubes (hemoglobin and hematocrit levels) or in dry tubes (creatinine, sodium, and potassium). Then, 30 mL of urine was collected from each of them. Blood and urine samples were analyzed immediately after collection. The creatinine assay was performed according to the Jaffé method [14], using a spectrophotometer RT-9200 (Rayto, Germany). Sodium [Na] and potassium [K] were measured by reference electron photometry, with an Electrolyte Analyzer ISE 4500 (Sfri, France), while hemoglobin [Hb] and hematocrit [Hct] were measured using an M-Series Automated Counter (Medonic, Sweden).

Serum creatinine (Screa) was introduced into the Chronic Kidney Disease Epidemiology Collaboration four-level race equation (CKD-EPI 4-level race) to assess the glomerular filtration rate (eGFR) [15]. eGFR values <90 mL/min/1.73 m^2^ were considered abnormal [16]. The fractional excretion of sodium (FeNa) was calculated according to the formula: FeNa (%) = 100 × (Urine Na [mmol/L] × Serum Crea [mg/dL])/(Serum Na [mmol/L] × Urine Crea [mg/dL]) [17]. Any value of FeNa > 1% was considered abnormal [17]. Serum potassium values <3.5 mmol/L were considered abnormal and suggested hypokalemia [18]. The fractional excretion of potassium (FeK) was calculated according to the following formula: FeK (%) = 100 × (Urine K [mmol/L] × Serum Crea [mg/dL])/(Serum K [mmol/L] × Urine Crea [mg/dL]) [19]. Any value of calculated urine Na/K ratio < 1 suggests acute functional renal failure [20]. Plasma volume variation (ΔPV) was determined using the Dill and Costill [21] formula based on [Hb] and [Hct] rates, as following: ΔPV (%) = 100 × (Hb_before_/Hb_after_) × [(1 − (Hct_after_/100))/(1 − (Hct_before_/100))] − 100, where Hb_before_ = hemoglobin before exercise, Hb_after_ = hemoglobin after exercise, Hct_before_ = hematocrit before exercise, and Hct_after_ = hematocrit after exercise. Any value of [Hb] < 12 g/100 mL was considered abnormal and suspected of anemia [22]. Pre-exercise (HRr), exercise (HRe) during training sessions, and maximal (HRmax) heart rates were measured with Accurex Plus cardiofrequencemeters (Polar, Finland). Pedometers Y-2028 (Decathlon Creation, China) were used to measure the distance each participant covered during the training sessions. The radiant or exposure temperature (T°) and relative humidity (RH) recorded during the training sessions were, respectively, 34.27 ± 3.47°C and 64.80 ± 7.52%. During training sessions and games, each player had to drink at least 1500 mL of tap water.

### 2.5. Statistical Analysis

The data were processed with Statistica software (Stat Soft Inc., version 12.0). The normality of the distribution of the variables was verified using the Kolmogorov–Smirnov test. The descriptive data were presented as mean (m) ± standard deviation (s). The one-way analysis of variance (ANOVA) for variables with normal distribution (eGFR, FeNa, Na/K, FeK, Crea, and urine Na and K) and Friedman's ANOVA (weight, BMI, body surface area, HR, Hb, and plasma Na and K) were used to compare M1, M2, and M3 measurements during the TC. In the case of significant ANOVA, the Tukey post hoc or Wilcoxon test was performed as appropriate for multiple comparisons. The significance level of the statistical tests was set at *P* < 0.05.

## 3. Results

The participants were 24 ± 6 years old with 165.33 ± 6.70 cm of height, 56.69 ± 5.88 kg of body weight, 46.80 ± 2.71 mL/min/kg of VO_2_max, and training average of 6 ± 1 hours per week for 11 ± 5 years. Their mean theoretical HRmax was 195 ± 5 bpm, and during the TC, the players trained at an average HR of 185 ± 9 bpm [170–204 bpm], or 94.38% of their theoretical HRmax. The distance covered per session during the TP was greater than that recorded during the IT (4198.50 ± 1426.51 m versus 2815.60 ± 642.19 m, *P* < 0.01). The minimum amount of 1500 mL of tap water was drunk per training session (IT and TP). No player had a personal or family history of kidney disease, hypertension, or diabetes. Nine participants were in the follicular phase, and six were in the luteal phase of the estrian cycle at the beginning of the study.

At the end of the IT (Table 1), the eGFR increased by 22.39% (*P* < 0.01), while the variation between the beginning and the end of TP was insignificant (*P* > 0.05). The eGFR variations recorded at the end of the IT were greater than those observed at the end of the TP (+22.39% versus −2.65%, *P* < 0.05). The number of players with an abnormally low eGFR (Table 2) was reduced from 11 to 5 (54.54%, *P* < 0.05) at the end of the IT and from 5 to 4 (*P* > 0.05) at the end of the TP.

FeNa increased by 143.85% (*P* < 0.01) at the end of the IT (Table 1), while the TP induced a reduction of 96.32% (*P* < 0.01). The number of women with a FeNa > 2% increased from 0 to 4 (*P* < 0.05) after IT, but at the end of the TP, the number decreased, respectively, from 4 to 0 for FeNa > 1% (*P* < 0.05) and >2% (*P* < 0.05). FeNa variations recorded at the end of the IT were greater than those observed at the end of the TP (+143.85% versus −96.32%, *P* < 0.001). At the end of the IT, the variation of FeK was not significant (*P* > 0.05), but the FeK increased by 144.41% (*P* < 0.001) at the end of TP, following the IT. The number of players with serum K < 3.5 mmol/L (Table 2) varied from 1 to 0 (*P* > 0.05) at the end of the IT and from 0 to 4 (*P* < 0.05) at the end of the TP. The urine Na-to-K ratio varied insignificantly (*P* > 0.05) at the end of IT as well as that of TP, and whatever the training phase, all female players had urine Na-to-K ratio > 1.

PV was reduced by 2.19%, and [Hb] has not changed at the end of the IT (*P* > 0.05). TP induced a decrease in PV of 17.45% and an [Hb] increase from 11.02 ± 0.83 g/100 mL to 12.10 ± 0.69 g/100 mL (+9.80%, *P* < 0.001) with a reduction in the number of players with an abnormally low value from 12 to 7 (*P* < 0.05). However, the variations were greater after TP for [Hb] (+9.80% versus +1.19%, *P* < 0.01) and PV (−17.45% versus −2.19%; *P* < 0.001) then IT.

## 4. Discussion

This study aimed at describing the changes in kidney parameters induced by 10 days of tapering (TP) during a training camp (TC) preparatory to a group competition, in 15 handball team members of Division 1 Amateur of Benin. During the tapering phase, the intensity was maintained, while the training frequency was reduced by 60%. This reduction in the training volume to maximize the positive effects of taper after an intensive training block is consistent with the literature [1, 2]. This is one of the first studies that provides information on the effects of tapering on renal parameters in athletes who are in the final stages of preparing for a competition. The results, however, are only valid for the players studied and cannot be generalized to all handball players in Benin, nor to those of other countries, without confirmation by subsequent work.

The study sample was of small size, and the parametric test used to analyze some parameters (e.g., Na/K) with a normal distribution, has not revealed significant differences, despite some large variations in the electrolyte levels. The further studies must include the measure of relevant plasma hormones (e.g., ADH and aldosterone), and analyzing the changes would provide valuable data to better understanding the findings related to hydroelectrolyte variations induced by training or exercise. The electrolyte intake and water consumption of the players after intensive training and tapering exercise were not assessed, but during the training sessions, they drank tap water (no sports drink ingested). These parameters must also be assessed in our following studies because electrolytes are highly affected by diet and beverages.

The eGFR increased at the end of the intensive training, which was maintained after tapering and confirmed by the reduction in the number of abnormal eGFR values. The variation in the eGFR is often attributed to hemodynamic changes, leading to an increase or decrease in renal blood flow [16]. Therefore, the increase in the eGFR can be considered as induced by hemodynamic adaptations, caused by the repetition of training sessions under high thermal stress. Repeated physical exercise, especially in hot climates, will reduce the effects of norepinephrine, a sympathetic neurotransmitter acting on the renal vascular system and, thus, induce an increase in blood flow to the kidneys [12]. This action is supported by decreased sympathetic activity and decreased plasma concentrations of angiotensin II and vasopressin, which are involved in reducing renal vasoconstriction [23]. The positive effect of the intensive training phase on renal function, particularly on glomerular function in participants, can, therefore, be associated with these neuroendocrine mechanisms.

The preservation of this positive effect during the tapering phase, despite a 60% reduction in the frequency of training sessions, indicates that the taper has helped to maintain the benefits of intensive training on glomerular function. Under these conditions, it is reasonable to believe that the intensity of training exercises is a determining factor in the improving glomerular function and maintaining it in good condition in athletes undergoing repeated stress. Studies will need to be carried out in the near future to understand the physiological mechanisms that support this improvement, by defining the limits of exercise intensities and the hydromineral conditions required. Repeated training sessions, conducted at low to moderate intensities, have been reported to result in less significant reductions in renal blood flow [12, 24], as well as less improvement in renal function in both sick and healthy individuals [25, 26]. In the reported studies, the hydromineral/hydroelectrolyte status of the participants was not documented. In this study, repeated exercise at high intensities still improved renal function in female handball players with a relatively low eGFR at rest. It must be recalled, however that normative values for athletes, particularly those from sub-Saharan Africa, remain to be determined [27].

FeNa increased at the end of the intensive training, but the values were between 1 and 2%. After the tapering, FeNa collapsed below 1%. The rise in FeNa at the end of the intensive training phase indicates an increase in sodium excretion by the kidneys, while its decrease at the end of tapering reflects sodium retention [17, 28]. The variation in FeNa depends on three main factors: glomerular filtration, tubular reabsorption and/or secretion, and sodium intake [29]. Therefore, the increase in FeNa can be attributed to the rise in the eGFR observed in female players but probably also to a high sodium intake. However, during the taper phase, FeNa decreased considerably below 1%. This FeNa value is typically in healthy subjects but suggests hypoperfusion of the kidneys often due to dehydration [30]. Dehydration is one of the causes of renal hypoperfusion associated with exercise. A decrease in the plasma volume resulting from dehydration affects kidney function and causes sodium retention with water by the kidneys, stimulated by the renin-angiotensin-aldosterone system and vasopressin to control plasma volume reduction [31, 32]. The considerable depletion of the plasma volume in our population at the end of tapering would then be the cause of the decrease in FeNa.

The FeK increased gradually from the end of the intensive phase to the end of the tapering, with normal values of serum potassium at each phase. FeK is a marker of potassium excretion by the kidneys, which reflects its secretion in the distal tubule, stimulated mainly by aldosterone, but also by the rate of plasma potassium [33]. Therefore, the rise in FeK throughout the training camp suggests an increase in potassium excretion by the kidneys, due to a large increased potassium secretion induced by aldosterone and/or high potassium intake. The implication of the former factor is more likely plausible because FeNa collapsed at the end of the camp. The increase in potassium excretion was confirmed by the number of cases of abnormally low values (<3.5 mmol/L) which varied from 0 to 4 players during tapering. It is, therefore, during this phase that potassium leakage was significant in some players. The high potassium excretion reflects also a transitory cellular adaptation to hypotonicity, due to stress (training sessions) and depending on the rate of plasma sodium decline [18, 34]. Urine potassium and FeK were very high, although the plasma potassium level was relatively low. It means that not only water intake but also potassium supplementation is essential.

The change in urine Na-to-K ratio was insignificant at the end of each of the two training phases but, throughout the camp, the urine Na-to-K ratio decreased to no less than 1. This decrease in the Na-to-K ratio confirms the consequence of dehydration on renal function. Under conditions of heat stress and hypohydration, aldosterone and vasopressin play key roles in maintaining homeostasis by promoting sodium and water retention [23]. As a whole, the training camp greatly disrupted the tubular function in this study. The elevated standard deviations for the FeNa and the urine Na-to-K ratio reflect a large interindividual variation. In addition, very large difference in the standard deviations for urine sodium and potassium reflects the group's heterogeneity on these electrolytes and may account for the results because this index is included. The dosage of these hormones, that is, vasopressin and aldosterone and the assessment evaluation of salt consumption would suggest a better explanation of this result.

The plasma volume gradually decreased from the intensive training phase (−2.19%) to the tapering (−17.45%), with a significant difference between the two phases. Repetition of intense training sessions under high heat stress, corresponding to temperatures at least equal to 34°C and relative humidity above 60%, could cause severe sweating, the means available to the body to evacuate excessive body heat and ensure cardiovascular stability [35]. The significant reduction in the plasma volume, therefore, reveals a poor compensatory hydration of the inevitable renal and sweat hydroelectrolytic losses, induced by intensive training and thermal stresses. The renin-angiotensin-aldosterone system will be activated and vasopressin release stimulated for both salt and water conservation by kidney function [31]. This decrease in the plasma volume indicates also hemoconcentration, confirmed by the increase in hemoglobin observed. Hemoconcentration is often caused in these conditions by a significant water loss by urine and sweating [30].

It is clear that the amount of water drunk by the players during the camp appears insufficient, given the significant reduction in the FeNa and plasma volume, and an increase in FeK. During the training sessions, players were encouraged to drink at least 1500 mL of tap water, but no recommendations were made during the recovery phase. However, data in the literature suggest that water intake equaling 150% of weight loss is required to optimize rehydration within 4 to 6 hours after exercise [36, 37]. The next work will have to take into account post-effort hydration and lead to consequent recommendations for athletes who practice in a hot environment. All those data recorded suggest that during the training camp, the handball players studied had inadequate hydration practice, especially during postexercise recovery. The results suggest the implementation of studies to define (1) the role of hydromineral regulation hormones in the renal response during training camps and (2) the influence of water status on the implementation of tubular reabsorption mechanisms in handball players under different practice conditions.

This study confirmed one of the positive effects of tapering, reported in the literature [2, 4]. It is the increase in hemoglobin levels during tapering. This increase is often attributed to a positive balance between hemolysis and erythropoiesis, stimulated by increased erythropoietin production [6]. In the current study, the rise of the hemoglobin rate observed may be a consequence of plasma volume depletion that induced hemoconcentration [30]. It may also be due to erythropoiesis, stimulated by the increased erythropoietin production during tapering, as highlighted by the increase in the number of red blood cells. Indeed, the number of red blood cells increased from 3.84 million cells/mcL to 3.90 million cells/mcL at the end of the intensive training phase, then to 4.55 million cells/mcL at the end of tapering.

## 5. Conclusion

Intensive training in a hot environment induced an increase in the eGFR in these female handball players, with a reduction in the number of abnormal values and then in the average fractional excretions of sodium (FeNa) and potassium (FeK). The tapering phase did not modify the eGFR but caused a reduction in FeNa and an increase in FeK. The urine Na-to-K ratio varied insignificantly at each phase of the training camp, but over the whole camp time, Na/K decreased. The intensive training phase, therefore, had a positive effect on the glomerular function of the players, whereas the tapering phase induced an incomplete recovery of the tubular function.

## Figures and Tables

**Table 1:** Anthropometric, hematological, physiological, and renal parameters of handball players during the training camp (*n* = 15).

	Measure 1	Measure 2	Measure 3
Weight (kg)	56.69 ± 5.88	57.98 ± 5.79^*∗∗*^	57.84 ± 6.23
Body mass index (kg/m^2^)	20.81 ± 2.62	21.30 ± 2.70^*∗∗*^	21.24 ± 2.78
Body surface area (m^2^)	1.64 ± 0.11	1.65 ± 0.11^*∗∗*^	1.65 ± 0.12
Heart rate before effort (bpm)	76 ± 7	67 ± 10^*∗∗∗*^	69 ± 10^*∗∗*^
eGFR (mL/min/1.73 m^2^)	84.01 ± 8.97	102.82 ± 20.31^*∗∗*^	100.09 ± 14.77^*∗∗*^
Fractional excretion of sodium (%)	0.57 ± 0.44	1.39 ± 0.98^*∗∗*^	0.05 ± 0.03^†††^
Fractional excretion of potassium (%)	2.17 ± 1.56	4.12 ± 1.78^*∗∗*^	10.07 ± 5.79^*∗∗∗*^^†††^
Urine sodium-to-potassium ratio	14.47 ± 12.88	8.54 ± 8.68	5.70 ± 5.31^*∗*^
Hemoglobin rate (g/100 mL)	10.89 ± 0.77	11.02 ± 0.83	12.10 ± 0.69^†††^^*∗∗∗*^
Serum creatinine (mg/dL)	1.07 ± 0.13	0.92 ± 0.12^*∗∗*^	0.92 ± 0.09^*∗∗∗*^
Serum sodium (mmol/L)	138.73 ± 4.81	141.93 ± 2.40	138.10 ± 1.79^†††^
Serum potassium (mmol/L)	4.46 ± 0.55	4.48 ± 0.36	3.62 ± 0.22^†††^^*∗∗∗*^
Urine sodium (mmol/L)	120.76 ± 70.03	94.20 ± 59.21	241.05 ± 145.41^†††^^*∗∗*^
Urine potassium (mmol/L)	14.50 ± 11.68	15.70 ± 13.18	59.71 ± 30.82^†††^^*∗∗∗*^
ΔPV (%)	−2.19 ± 6.97	−17.45 ± 7.55^*∗∗∗*^	−19.54 ± 6.98^*∗∗∗*^

The values represent the means ± standard deviations; *n*: sample size; Measure 1: performed at the beginning of intensive training; Measure 2: performed 36 hours after the end of intensive training; Measure 3: performed 36 hours after the end of tapering; eGFR: estimated glomerular filtration rate with the of Chronic Kidney Disease Epidemiology equation [15]; ΔPV: plasma volume variation; Measure 1 of ΔPV: variation between the measure before and at the end of intensive training; Measure 2 of ΔPV: variation between the measure at the end of intensive training and the end of tapering; Measure 3 of ΔPV: variation between the measure before and the end of the whole training camp; ^*∗*^: difference with Measure 1, significant at *P* < 0.05; ^*∗∗*^: difference with Measure 1, significant at *P* < 0.01; ^*∗∗∗*^: difference with Measure 1, significant at *P* < 0.001; ^†††^: difference with Measure 2, significant at *P* < 0.001.

**Table 2:** Frequency of abnormal values of renal parameters of handball players during training camp (*n* = 15).

	Measure 1 Number (%)	Measure 2 Number (%)	Measure 3 Number (%)
eGFR (mL/min/1.73 m^2^)			
>90	4 (26.66)	10 (66.66)^*∗*^	11 (73.33)^*∗∗*^
<90	11 (73.33)	5 (33.33)^*∗*^	4 (26.66)^*∗∗*^
Fractional excretion of sodium (%)			
<1	12 (80)	7 (46.66)	15 (100)^*∗∗*^^††^
>1	3 (20)	4 (26.66)	0 (00)^*∗*^^†^
>2	0 (00)	4 (26.66)^*∗*^	0 (00)^†^
Serum potassium (mmol/L)			
<3.5	01 (06.66)	0 (00)	04 (26.66)^†^
>3.5	14 (93.33)	15 (100)	11 (73.33)^†^
Hemoglobin rate (g/100 mL)			
>12	1 (06.66)	3 (20)	8 (53.33)^*∗∗*^^†^
<12	14 (93.33)	12 (80)	7 (46.66)^*∗∗*^^†^

The values represent the absolute and relative frequencies of the abnormal values of the renal parameters studied; *n*: sample size; Measure 1: performed at the beginning of intensive training; Measure 2: performed 36 hours after the end of intensive training; Measure 3: performed 36 hours after the end of tapering; eGFR: estimated glomerular filtration rate using the Chronic Kidney Disease Epidemiology formula [15]; ^*∗*^difference with Measure 1, significant at *P* < 0.05; ^*∗∗*^difference with Measure 1, significant at *P* < 0.01; ^†^difference with Measure 2, significant at *P* < 0.001; ^††^difference with Measure 2, significant at *P* < 0.01.

## Data Availability

The data used to support the findings of this study are available from the corresponding author upon request.
